# Hybrid Multi-Label Classification Model for Medical Applications Based on Adaptive Synthetic Data and Ensemble Learning

**DOI:** 10.3390/s23156836

**Published:** 2023-07-31

**Authors:** M. Priyadharshini, A. Faritha Banu, Bhisham Sharma, Subrata Chowdhury, Khaled Rabie, Thokozani Shongwe

**Affiliations:** 1 Department of Computer Science Engineering, Nalla Malla Reddy Engineering College, Hyderabad 500088, Telangana, India; priyadharshini.cse@nmrec.edu.in; 2 Department of Computer Science, Karpagam Academy of Higher Education, Coimbatore 631027, Tamil Nadu, India; farithabanu.ahamedsheriff@kahedu.edu.in; 3 Chitkara University Institute of Engineering and Technology, Chitkara University, Rajpura 140401, Punjab, India; 4 Department of Computer Science and Engineering, Sreenivasa Institute of Technology and Management Studies, Chittoor 517127, Andra Pradesh, India; subrata895@gmail.com; 5 Department of Engineering, Manchester Metropolitan University, Manchester M15GD, UK; 6 Department of Electrical and Electronic Engineering Technology, University of Johannesburg, Auckland Park, Johannesburg 2006, South Africa; shongwe@uj.ac.za

**Keywords:** imbalanced data, adaptive synthetic data, improved particle swarm optimization, adaptive neuro-fuzzy inference system, probabilistic neural network, multi-class classification

## Abstract

In recent years, both machine learning and computer vision have seen growth in the use of multi-label categorization. SMOTE is now being utilized in existing research for data balance, and SMOTE does not consider that nearby examples may be from different classes when producing synthetic samples. As a result, there can be more class overlap and more noise. To avoid this problem, this work presented an innovative technique called Adaptive Synthetic Data-Based Multi-label Classification (ASDMLC). Adaptive Synthetic (ADASYN) sampling is a sampling strategy for learning from unbalanced data sets. ADASYN weights minority class instances by learning difficulty. For hard-to-learn minority class cases, synthetic data are created. Their numerical variables are normalized with the help of the Min-Max technique to standardize the magnitude of each variable’s impact on the outcomes. The values of the attribute in this work are changed to a new range, from 0 to 1, using the normalization approach. To raise the accuracy of multi-label classification, Velocity-Equalized Particle Swarm Optimization (VPSO) is utilized for feature selection. In the proposed approach, to overcome the premature convergence problem, standard PSO has been improved by equalizing the velocity with each dimension of the problem. To expose the inherent label dependencies, the multi-label classification ensemble of Adaptive Neuro-Fuzzy Inference System (ANFIS), Probabilistic Neural Network (PNN), and Clustering-Based Decision tree methods will be processed based on an averaging method. The following criteria, including precision, recall, accuracy, and error rate, are used to assess performance. The suggested model’s multi-label classification accuracy is 90.88%, better than previous techniques, which is PCT, HOMER, and ML-Forest is 65.57%, 70.66%, and 82.29%, respectively.

## 1. Introduction

Multi-label classification is typically used in different data mining applications, like labeling videos, images, music, and texts. Multi-label classification classifies documents into various classes simultaneously based on their properties. This task is different from the conventional single label, which correlates every document to a single class. The classification task of the single label can also be regarded as multi-class or binary classification [1]. In multi-class classification, every document can fall under multiple label categories, but only one label category is designated. However, the multi-label classification, generalizes the multi-class and binary classification since it does not emphasize any constraints on the number of components that are imposed on the outputs [2]. Techniques of multi-label classification are affected by a higher level of class imbalance, and due to this, they will not be able to operate with efficiency.

Multiple item identification in images, gene expression prediction, tag prediction for audio, and categorizing papers into specified categories are just a few examples of the many fields where multi-label classification is a common challenge in artificial intelligence research [3,4]. Additionally, classifiers that can develop a global model for a class perform worse when multi-label datasets are classified [5]. To concentrate on the decision limits of the classifier(s) in each area, clustering has previously been used to extract the distribution of data completely or separately for each class [6,7]. In other words, one classifier is trained for each cluster of data after the initial clustering of the data. The labels of the data are not used in clustering, which is an unsupervised classification [8,9]. There is no method to collect the relationship between labels since many algorithms that address the multi-label classification issue neglect the connection between labels.

Instead of looking at labels separately, using the correlation between labels may help with multi-label categorization problems. The first Bi-cluster based Synthetic Minority Oversampling Technique (B-SMOTE) for managing classification with unbalanced datasets was presented in recent work as a solution to these problems. Moreover, the BAT optimization algorithm is used for feature selection, which selects more relevant features from the samples to increase the multi-label classification accuracy. Then, an ensemble classifier is used for classification. In tasks requiring many labels, the hierarchical model may thus be particularly effective. However, in existing work, SMOTE is used for data balancing in which SMOTE does not take the enclosing instances coming from various classes into consideration during the generation of synthesized examples. As a result, there may be more class overlap and more noise introduced. An innovative strategy was used in this study to eliminate these problems called adaptive synthetic data-based multi-label classification.

### Organization of This Research Work

In this research work, an introduction to the multi-label classification is given in Section 1. Multi-label categorization research is reviewed in Section 2. In Section 3, the proposed multi-label classification model design is formulated, and then a closed-form solution is derived. Section 4 discusses the results of the simulation. The conclusion and work intended for the future are discussed in Section 5.

## 2. Literature Review

This section reviews the multi-label classification with different methods in detail.

Mikolov et al. [10] the Recurrent Neural Network Language Model’s Contextual Real-Valued Input Vector for Every Word, which enhances performance. However, there are a few drawbacks to the techniques at varied levels. On the usage of the CNN technique, post the calculation of convolutions, k-max or average pooling must still be used to determine the length of each document’s representation. The RNN model is biased, with later words dominating earlier words.

Lai et al. [11] suggested the RCNN model, which can overcome RNN bias, for document categorization. While learning the feature representation of texts using CNN, the recurrent structure’s benefits are successfully used by the RCNN, which facilitates the gathering of contextual data since this approach will also encounter problems as a result of vanishing gradients and exploding gradients while the error is updated with back-propagation.

Lin et al. [12] proposed that Label Space Dimension Reduction (LSDR) is performed using a unique technique called End-to-End Feature-Aware Label Space Encoding (E 2FE). In an early study, E 2FE learns a code matrix directly from training example code vectors without an encoding algorithm. The coding matrix is obtained by concurrently optimizing the restoration and the predictability of the latent space, which explains E2FE feature awareness. E2FE additionally teaches a linear decoding matrix to quickly find an unknown instance’s label vector from its estimated code vector. E2FE trains predictive models to convert instance attributes into code vectors according to the learned code matrix.

The multi-label categorization must be handled, and an enhanced Convolutional Neural Network through Hierarchical Dirichlet Process (HDP) model has been put out by Wang et al. [13] for issues in NLP. An HDP model is applied first to exclude a few less semantically significant terms. After that, convert words into vectors using word-embedding techniques. The last step is to train CNN using word vectors. In terms of performance, experimental findings show that our technique outperforms both text CNN and the majority of conventional multi-label classification algorithms.

A multi-label ranking approach for document categorization based on LSTM was suggested by Yan et al. [14], specifically LSTM22, which combines a unified learning-ranking approach called rank LSTM with rep LSTM, an adaptive data representation process. The supervised LSTM in repLSTM incorporates the document labels to learn document representation. Our method also performs well in document categorization tests on three representative datasets.

Multi-label text may now be effectively and automatically classified using a revolutionary technique given by Jindal [15]. Lexical and semantic ideas serve as the foundation for the suggested approach. A common IEEE taxonomy is used to identify tokens in text sources. Using the well-known lexical database WorldNet, the semantic links between tokens are examined. A database of 150 computer science research studies from the IEEE Xplore digital library is used to test the suggested technique. With a 75% accuracy rate, it has shown noticeably excellent performance.

The greatest resampling methods, such as random oversampling, heuristic under-sampling, and synthetic sample generation approaches, were combined in a suggested strategy by Charte et al. [16]. To understand how the label decoupling procedure affects the behavior of these hybrid approaches, empirical analysis is performed. Therefore, the experimentation may provide a remarkable set of recommendations for combining various strategies.

Latent class models combined with novel algorithms in classification were proposed by Alyousef et al. [17]. The new approach clusters patients into groups using latent class models, which enhances classification and makes it easier to identify the fundamental distinctions between the groups that are found. Data from individuals with Systemic Sclerosis, an uncommon and possibly deadly illness, are used to evaluate the approaches. Results reveal that when compared to competing comparable approaches, accuracy is improved using the “Latent Class Multi-Label Classification Model”.

An AC*k*EL paradigm was introduced by Wang et al. [18]. Based on active learning, a label-selection condition assesses class differentiability and balance. Subsequently, the suggested condition is used to randomly choose the first label or label subset and iteratively select the others. ACkEL uses pool-based and stream-based models in disjoint and overlapping modes, respectively. Comparing the techniques shows that they are possible and successful.

An innovative approach referred to as FL-MLC, Che et al. [19] suggested a method for multi-label learning that takes feature–label interdependence considered. The intrinsic link between the feature variable and the label variable is originally presented as the discriminant weight of any feature to label. The label’s feature distribution for inputs illustrates the distinctive weights of characteristics; kernel alignment and multiple kernel learning increase computation. The feature distribution-based label correlation uses two aggregation processes to combine feature distributions on various labels. The random label variables with almost identical feature distributions should be carefully considered. The feature distribution-based label correlation is used in this way to alter the distance among the parameters for various labels in the FL-MLC approach’s predictive learner. Finally, it can be shown from the results of the tests performed on twelve real-world datasets that the method produces improved multi-label classification results in terms of efficiency and variety.

An innovative classification margin-based MNRS model was presented by Sun et al. [20]. A filter-wrapper pre-processing strategy for feature selection utilizing the modified Fisher score model reduces the spatiotemporal complexity of multi-label data and improves classification performance. It is confirmed from the results of experiments conducted that the suggested technique with thirteen multi-label datasets is efficient in the selection of salient features, showing its remarkable classification potential in multi-label datasets.

Huang et al. [21] proposed a novel BLS-MLL multi-label classifier with two new mechanisms: correlation-based label thresholding and a kernel-based feature-reduction module. The feature-mapping layer, enhancement-nodes layer, and feature-reduction layer are the three levels that make up the kernel-based feature-reduction module. Elastic network regularization solves feature randomness to increase performance at the feature-mapping layer. For efficient high-dimensional nonlinear conversion in the enhancement-nodes layer, the kernel approach is used. BLS-MLL may build a label-thresholding function to convert final decision values to logical outputs using correlation-based label thresholding, boosting classification performance. Eventually, on ten datasets, BLS-MLL is superior to six cutting-edge multi-label classifiers. BLS-MLL beats the comparable algorithms in 86% of instances and has greater training efficiency in 90% of cases, according to classification performance findings.

Bayati et al. [22] suggested a subspace learning-based memetic method for global and local search in multi-label data. For multi-label feature selection, this is the first attempt using a filter-based memetic algorithm. Reconstruction error and sparsity regularization are the objective function’s conflicting objectives. The suggested multi-label feature-selection approach is compared against nine filter-based methods. Classification accuracy, hamming loss, average precision, and one mistake are used to evaluate multi-label classification performance. The suggested strategy outperforms comparable methods across all assessment criteria in eight real-world datasets.

Zhu et al. [23] suggested Multi-Label Classification with Dynamic Ensemble Learning (MLDE), an innovative approach. MLDE predicts each unseen occurrence with the most competent ensemble of base classifiers. Classification accuracy and ranking loss as basis classifier competency measurements to create dynamic choices for the multi-label issue and improve performance. Classification accuracy is decomposable to numerous labels and differentiates a classifier’s ability to distinguish labels, whereas ranking loss focuses on a classifier’s total performance on the label set and completely analyses the connection among many labels. In comprehensive tests on 24 publicly available datasets, MLDE exceeds modern techniques.

Zhang et al. [24] suggested Relief-LIFT, a multi-label classification approach. Relief-LIFT uses LIFT to produce new features, then changes Relief to choose informative features for the classification model. Our Relief-LIFT approach outperforms previous multi-label classification algorithms on 12 real-world data sets. Table 1 shows the comparative analysis between the available techniques of multi-label classification.

### 2.1. Contribution to the Work

The main contributions of this work are:

(i) Adaptive synthetic sampling-based synthetic data generation is performed to balance the input data class label;
(ii) Data normalization is used to normalize the input data scale based on min-max normalization, which increases the accuracy of the proposed classification model;
(iii) Significant features are selected based on avoiding redundant and noisy data from the samples using velocity-equalized particle swarm optimization. Standard particle swarm optimization has the issue of early convergence, and it has been strengthened by balancing the velocity (v) with each aspect of the issue in the proposed velocity equalized particle swarm optimization model;
(iv) Multi-label classification using an ensemble of Adaptive Neuro-Fuzzy Inference Systems, Probabilistic Neural Networks, and Clustering-Based Decision Tree methods processed based on averaging method.

### 2.2. Motivation

Multi-Label Classification (MLC) has many applications, such as bio-informatics as diseases stages identification; text classification, e.g., politics, the environment, and economics are among the categories in which news articles can be categorized; sentiment evaluation of microblogs, for instance, can classify a microblog’s emotional state into two or more categories, including joyful, furious, worried, and surprised; system of suggestions. Movie grouping, for example, a single movie may be related to more than one genre, such as action, romantic, and thriller; multi-disease risk identification, for example, one individual might suffer from yeast disease, diabetes, and other diseases; and book recommender, for example, could suggest numerous books for a reader; journal article grouping, for example, a journal paper may fall under various groups, such as multi-label classification, feature selection, filter method, and wrapper method. In all the applications involving multi-label classification, identifying the interdependence of labels, feature selection, handling imbalances, or any combination of these is a crucial and fundamental issue. These issues should be well resolved to obtain higher prediction performance in real-world applications. These challenges motivate us to develop new MLC methods that provide the desired results for the respective applications.

## 3. Proposed Methodology

**Figure 1:**
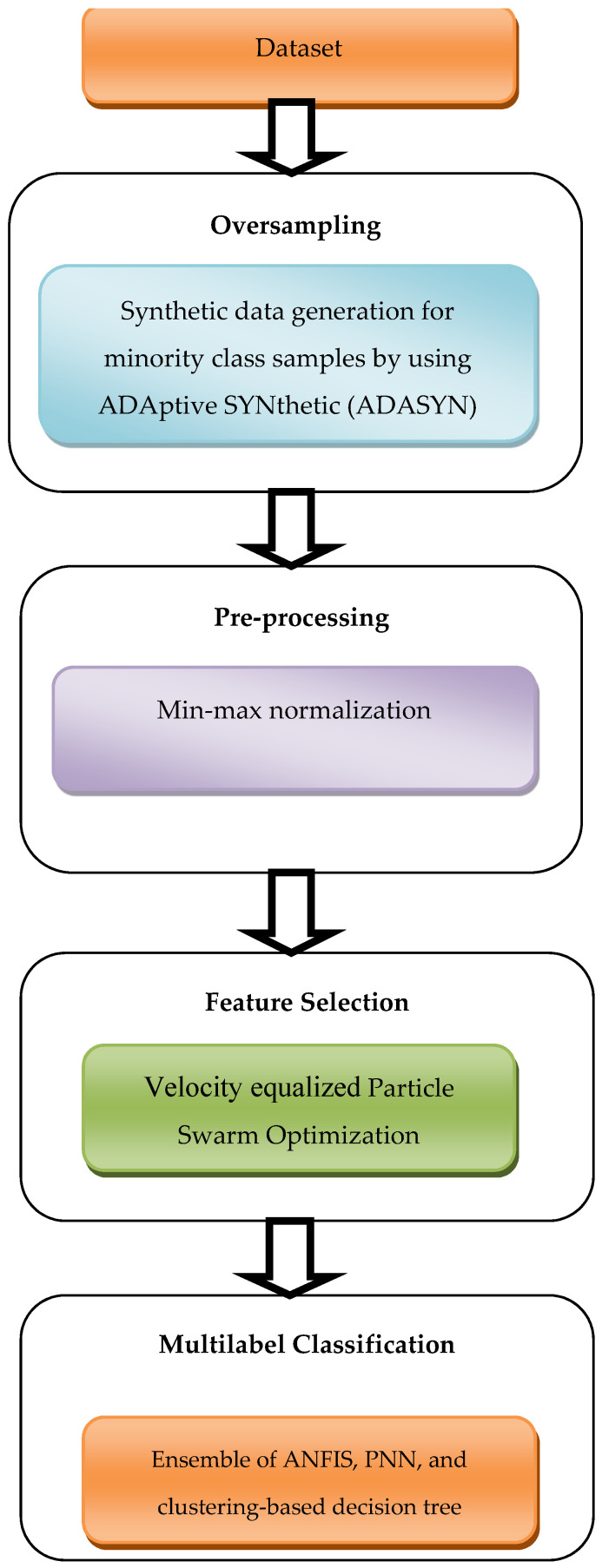
Block architecture of the proposed work.

This section explains the proposed improved multi-label classification model. It consists of four modules first one is pre-processing using min-max normalization; the second one is an ADAptive SYNthetic (ADASYN) sampling strategy for studying unbalanced data sets; the third one, improved PSO, is used for feature selection that selects additional pertinent traits from the samples, the Ensemble approach, which comes in at number four, is the one that is employed for multi-label categorization. Figure 1 depicts the overall architecture of the suggested work.

### 3.1. Data Balancing Using ADAptive SYNthetic (ADASYN) Sampling

ADASYN (Algorithm 1) sampling is used in this work to oversample using artificial samples created using the following process on the minority class [25,26]:
**Algorithm 1: ADASYN**  Step 1: Start.  Step 2: Input Training data set $D_{tr}$ having m samples $\left\{x_{i},y_{i}\right\},i=1,...,m,$ where $x_{i}$ represents a particular occurrence of the features X and in the n-dimensional feature space $y_{i}\in Y=\{1,-1\}$ is $x_{i}$ assigned the class identity label. According to this definition, the percentage of minority class cases and majority class examples is represented by the letters ms and ml correspondingly. Hence, $m_{s}\leq m_{l}\;and\;m_{s}+m_{l}=m.$  Equation (1) computes the degree of class imbalance: (1)$$d=\frac{m_{s}}{m_{l}}$$
where d∈(0,1]  Step 3: If $d<d_{th}$ then $d_{th}$ is the current cut-off point for the proportion of class unbalance that may be accepted. 
(a)Determine the required number of samples for the minority class; synthetic data included is given in Equation (2);
(2)$$G=\left(m_{1}-m_{s}\right)\times \beta$$
where β∈[0,1], following the creation of the synthetic data, is a parameter considered to establish the intended level of balance. β=1 signifies that after the generalization process, a completely balanced data set is produced;
(b)For each example $x_{i}\in minority\;class$, calculate the ratio $r_{i}$ as in Equation (3). It entails identifying K’s nearest neighbors in n-dimensional space using the Euclidean distance;

(3)$$r_{i}=\frac{{∆}_{i}}{k_{}}\;i=1,\ldots m_{s}$$
where $\Delta_{i}$ is how many instances in xi’s K closest neighbors fall into the majority class, hence $r_{i}\in [0,1];$
(c)Normalize $r_{i}by\hat{r_{i}}=r_{i}/\sum_{i=1}^{m_{s}}r_{i},$ so that $\hat{r}$ I provides a distribution of density ($\sum_{i=1}^{m_{s}}r_{i}$ = 1);(d)Determine how many instances of synthetic data must be produced for each minority example in xi according to Equation (4);
(4)$$g_{i}=\hat{r}{}_{i}\times G$$
  G is the number of synthesis data samples that must be created for the minority class by the definition given in Equation (2);
(e)Example data for every minority class xi, create gi examples of synthesized data using the procedures below:
  Do the loop from 1 to gi:   Choose one minority, then choose a random data value, xzi, the K closest neighbors for data xi.  Create the synthetic data, $s_{i}$ as in Equation (5), for instance:
(5)$$s_{i}=x_{i}+\left(x_{zi}-x_{i}\right)\times \lambda$$
where (xi − xi) in n-dimensional spaces forms the difference vector, and λ stands for a random number: λ∈[0,1].  End Loop  Step 4. End.

Utilizing a density distribution is the main principle of the ADASYN algorithm $\hat{r}{}_{i}$ as a factor in determining how many synthesized samples must be created for every minority data example. Physically, $\hat{r}{}_{i}$ indicates an assessment of the weights that are assigned to various minority class instances based on how tough they are to learn. The final dataset after ADASYN will drive the learning algorithm to concentrate on the cases that are challenging to learn while simultaneously providing a balanced representation of the data distribution.

### 3.2. Pre-Processing Using Min—Max Normalization

The data was scaled in this method to a range of [0, 1] or [−1, 1]. Algorithm 2 produces the detailed steps of this model. This method applies the formula to transform the input value of the attribute X from [low, high] to x norm by using Equation (6).

(6) $$x_{norm}=\frac{\left(high-low\right)*\left(x-minX\right)}{maxX-minX}$$

where minX and maxX are the lowest and maximum values for the incoming data set’s property X. The data is scaled using the method to a range of [0, 1], and it is then sent to the feature selection stage [27,28].
**Algorithm 2: Min-Max normalization**Input: Pima Indian, Yeast 1, and New—thyroid 1 datasets Output: Normalized values for all datasets Step 1: Start Take the maximum value from the array. Step 2: Take the minimum value from the array. Step 3: Estimate and show the average value from the array and the number of values that are larger than the average. Step 4: Estimate and show the normalized values of the original array values using Equation (6). Step 5: End.

### 3.3. Feature Selection Using Velocity Equalized Particle Swarm Optimization

After data normalization, select features to reduce the time consumption. This work uses Particle Swarm Optimization (PSO) for feature selection.

#### 3.3.1. Particle Swarm Optimization

PSO computation uses a swarm of particles that each represents a solution contender. The system is initialized with a population that is made up of random solutions, upgrading its generational data to identify optimum solutions. The search procedure makes use of a combination of deterministic and probabilistic principles, both of which are dependent on the members of the population exchanging information with one another to make the procedure more effective. On the other hand, PSO does not include any evolution operators such as crossover or mutation. Using their independent memory and the information that the swarm has collected, each particle in the search space develops a proposed solution over time. In PSO, the information sent along from particle to particle is limited to the ranking of the global best particle obtained from the swarm. In most cases, it is a strategy for transferring information in just one direction. PSO particles rapidly converge to the best solution, reducing computation time [29].

However, PSO has the issue of early convergence, and it easily falls into the local optimum solution. To overcome those issues, this work introduced the velocity equalized particle swarm optimization.

#### 3.3.2. Velocity Equalized Particle Swarm Optimization

##### Initial Population

Modeling the feature-selection issue mathematically, finding the ideal subset containing useful features is the goal to be achieved by the feature selection problem, which is described as an optimization problem [30].

A vector is used to represent a collection of yeast traits given by F,Fi=fi,1,fi,b,…,fi,j,…,fi,t, where t is the overall number of yeast traits, and i represents the number of data samples. FS should be provided with a fresh set of educational features $SF_{i}=s_{i},1,s_{i},2,\ldots ,s_{i},j,\ldots ,s_{i},m,$ this has a new length m that the selection process generates, $s_{ij}\{0,1\},j=1,2,\ldots ,m.$ If $s_{o,j}=1$ signifies that the sample number has chosen the jth feature as an informative feature i. But, if $s_{}$o, j = 0 indicates that the jth yeast characteristic in the data sample number i is either concealed or uninformative.

##### Solution Representation

Each solution (particle) represents a subset of sample characteristics in the VPSO method for the selection issue. The VPSO swarm consists of several particles (positions), each of which contains one or more positions, expressed as binary vectors in a row (features). Every point in the sample corresponds to a feature. The situation of the particle is indicated by its location feature.

The VPSO algorithm’s feature selection process starts with random solutions and refines the population to get the best overall solution. An entirely new subset of the sample characteristics is created by the best solution. In the supplied database, each distinct characteristic is regarded as a separate search area. Table 1 displays the feature selection technique’s solution representation.

When position j is equal to 1, the jth feature is chosen in the form of an informative feature; when it is equivalent to 0, it is not selected. The jth feature is not included in the original text if the position j is equal to 1 [31].

##### Fitness Function

Each potential solution provided by the feature selection algorithms is assessed using the Fitness Function (FF), which serves as a measurement of effectiveness. The fitness function for each generation is calculated for all potential solutions. This solution will be swapped out for the present one if the quality of the solution is improved, and vice versa. The VPSO method provides a solution to the feature selection problem for the present document that is thought to have a high fitness function value. In this study, the fitness function for the feature selection issue was the classification accuracy in the VPSO method. The algorithm may easily slip in the local minimum since the typical VPSO has the issue of premature convergence.

##### Position Updation

VPSO updates each particle’s location depending on two key factors: velocity and particle position. Each particle seeks to migrate to the ideal place by updating its velocity based on the influence of particle movement.

The suggested method addresses the issue of early convergence and has been strengthened by balancing the velocity (v) with each aspect of the issue. Each population dimension has a unique factor v given to it. Therefore, exploration and exploitation procedures are carried out concurrently for each dimension.

In VPSO, the best solution’s j-dimensional local search space is used. Equation (7) is updated up to dimension j as follows, doing a local search around the best solution in VPSO by using Equation (7).

(7) $$v_{ij}^{t+1}=\left\{\begin{array}{c} {\alpha v}_{ij}^{t}{xand}_{j}>x_{ij}\\ vij{}^{t}otherwise, \end{array}\right.$$

Based on the number of iterations, the inertia weight value often varies between [0, 1]. As of iteration number I, LBI is the best local solution available, while GBI is the best global solution. r and 1, r and 2, and c1 and c2 are typically two constants, whereas Rand is a random integer between [0, 1]. Equation (8) establishes the inertia weight.

(8) $$W=\left(w_{max}-w_{min}\right)\times \left(\frac{I_{max}-I}{I_{max}}\right)+w_{min}$$

where $w_{}{}_{max\;and\;lumin}$ are, respectively, the greatest and smallest inertia weights. These weights have values that are fixed and fall between 0.5 and 0.9. Algorithm 3: produces the step-by-step procedure for improved particle swarm optimization.
**Algorithm 3: Velocity equalized particle swarm optimization**Step 1: Start. Step 2: Swarm (job initialization) randomly initialize the position and velocity of each particle. Step 3: Particle (feature) fitness (classification accuracy) evaluation if the fitness of *xi* > *pbesti* *pbesti* = *xi* if the fitness of *pbesti* > *gbesti* *gbesti* = *pbesti* Step 4: Update the velocity of particle (feature) i
$$v_{ij}^{t+1}=\left\{\begin{array}{c} {\alpha v}_{ij}^{t}{xand}_{j}>x_{ij}\\ {vij}^{t}otherwise, \end{array}\right.$$
Update the position of the particle (feature) *i*$V_{id}^{t+1}$
$$x_{id}^{t+1}=x_{id}^{t}$$
Step 5: If the halting requirement is not satisfied, go to Steps 2 and 3. Step 6: Return *gbest* and its *fitness values (classification accuracy)*. Step 7: End. 

### 3.4. Multi-Label Classification Using Ensemble Classification

After feature selection, those selected features are sent to the classifier for multi-label classification. This work uses an Ensemble of Adaptive Neuro-Fuzzy Inference Systems, Probabilistic Neural Networks, and Clustering-Based Decision Tree methods for classification. Ensemble learning is performed by averaging the model.

#### 3.4.1. Adaptive Neuro-Fuzzy Inference System (ANFIS)

The selected features are given to the ANFIS as input. One sort of neural network that operates on the neuro-fuzzy network is the ANFIS network. All the nodes on the top layer are adaptable nodes. The fuzzy membership grade of the inputs is layer 1’s outputs [32,33].

The nodes are fixed nodes in the second layer. They have the letter M written on them, suggesting they function as a straightforward multiplier. These are possible representations of this layer’s outputs. The nodes are likewise fixed nodes on the third layer. They are marked with the letter N, indicating that they normalize the firing intensities from the preceding layer. The nodes are adaptive in the fourth layer. Simply the normalized firing intensity and a first-order polynomial are combined to produce each node’s output in this layer. Only one permanent node with the letter S is present in the fifth tier. The summing of all incoming signals is done by this node.

#### 3.4.2. Probabilistic Neural Network

A Probabilistic Neural Network is made up of numerous sub-networks, each of which is an estimate of the pdf for a particular class [34]. Input data are included in the input nodes. With the train set’s points serving as centers, the second layer is made up of Gaussian functions. The outputs from the second layer for each class are averaged in the third layer. Voting is done at the fourth tier, and the highest value is chosen [35,36].

#### 3.4.3. Clustering-Based Decision Tree

Training data set $(X_{i}{,Y}_{i}),i=1,2,3,\ldots \ldots ,N$ where Xi refers to a continuous-valued vector in n dimensions, and $Y_{i}=\{0,1\}$ with “0” for normal and “1” for abnormality denotes the matching class designation. Training and testing are the first and second phases of the suggested procedure [37]. The training space is divided into k distinct clusters $C_{1},C_{2},C_{3},\ldots ,C_{K}$ by using steps 1–3 of the k-Means-based anomaly identification approach. The cases in each k-Means cluster are then used to train the C4.5 decision tree. Every training instance is just connected to a single cluster according to the k-Means approach. But, the C4.5 decision tree, whose training is done on that cluster fine, tunes the decision boundaries by dividing the instances using a set of if-then rules across the feature space when any subgroups or overlaps are present inside a cluster.

#### 3.4.4. Ensembling

**Figure 2:**
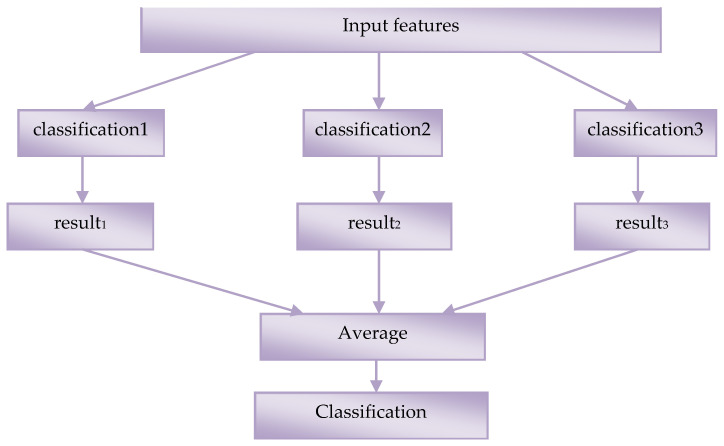
Ensemble Learning. The steps involved in this method are, to create N experts with individual starting points. 1. Train every expert individually. 2. The values of the experts were combined and averaged.

All ensemble-based systems are founded on the premise that merging multiple models may lower the error via averaging since distinct classifiers or features might produce different mistakes. When a single model is unable to do these tasks, ensemble learning is typically utilized to increase classification or prediction performance, particularly when dealing with multi-class situations. The ensemble is done by averaging the output of individual networks, shown in Figure 2.

## 4. Results and Discussion

Compare the proposed ASDMLC’s performance against that of the PCT, Hierarchy of Multi-label Classifier (HOMER), A ML-FOREST, BARF-MLC, and FSEA-MLC techniques in this experiment in terms of precision, recall, accuracy, and f-measure are calculated and evaluated with a 10-fold cross-validation procedure, and its average values are given in below Tables [38,39]. The utilized datasets are detailed below. Name, instance count, attribute type for each data collection, and the imbalance ratio value. According to Table 2, each data file is organized as follows.

**Feature Selection results:**

**1. Pima Dataset:**

Totally 6 Features are selected as follows

Pregnancies, Glucose, Blood Pressure, Insulin, BMI, Age.

**3. Yeast Dataset:**

Totally five features were selected as follows,

Mcg, gvh,alm,mit,erl.

**Description:**

Feature 1. mcg: mcg feature describes McGeoch’s approach for signal sequence recognition.

Feature 2. gvh: It describes von Heijne’s technique for signal sequence recognition.

Feature 3. alm: It describes the Score of the ALOM membrane-spanning region prediction program.

Feature 4. mit: It describes the Score of discriminant anatomy of the amino acid content in the N-terminal region of mitochondrial and non-mitochondrial proteins.

Feature 5. erl: It describes the Presence of the “HDEL” substring Binary attribute.

**3. Thyroid Dataset:**

Totally four features are selected as follows,

T3resin, Thyroxin, Triiodothyronine, TSH_value.

### 4.1. Experimental Setup and Comparative Analysis

The experimental setup is shown in Table 3, and the performance comparison findings are shown in Table 4, Table 5 and Table 6.

Experimental setup for the proposed multi-label classification model, such as software used and the number of data used for training and testing, are clearly given in Table 3.

### 4.2. Performance Metrics

(1) Precision

Precision is a percentage of outcomes that are significant and is described as

(9) $$\mathrm{Precision}=\frac{Truepositive}{truepositive+falsepositive}$$

(2) Recall

It is described as the percentage of all relevant results that the suggested algorithm classifies as relevant, and it is as follows:

(10) $$\mathrm{Recall}=\frac{Truepositive}{truepositive+FalseNegative}$$

(3) Accuracy

The percentage of correct predictions made by this algorithm is called accuracy, as shown in the following:

(11) $$\mathrm{Accuracy}=\frac{Truepositive+TrueNegative}{Total}$$

(4) F measure

An F-score is the harmonic mean of a system’s precision and recall values.

2 × [(Precision × Recall)/(Precision + Recall)] (12)

### 4.3. Comparison of Proposed and Existing Models

Table 4 produces the numerical results for the proposed ASD-MLC and existing PCT, HOMER, ML-FOREST, BARF-MLC, and FSEA-MLC models of the yeast dataset in terms of accuracy, precision, recall, and f-measure. From the above table, it is proven that the proposed ASD-MLC obtains better results in all metrics, such as accuracy, precision, recall, and f-measure, than the existing PCT, HOMER, ML-FOREST, BARF-MLC, and FSEA-MLC models. For example, the proposed ASD-MLC achieves 90.23% accuracy, and the existing PCT, HOMER, ML-FOREST, BARF-MLC, and FSEA-MLC models achieve 69.53%, 72.26%, 83.07%, 87.23%, and 88.67%.

Table 5 shows the numerical results for the proposed ASD-MLC and existing PCT, HOMER, ML-FOREST, BARF-MLC, and FSEA-MLC models of the Pima dataset in terms of accuracy, precision, recall, and f-measure. From the above table, it is proven that the proposed ASD-MLC obtains better results in all metrics, such as accuracy, precision, recall, and f-measure, than the existing PCT, HOMER, ML-FOREST, BARF-MLC, and FSEA-MLC models. For example, the proposed ASD-MLC achieves 90.88% accuracy, and the existing PCT, HOMER, ML-FOREST, BARF-MLC, and FSEA-MLC models achieve 65.56%, 70.65%, 82.29%, 86.71%, and 88.67%.

Numerical results for the proposed ASD-MLC and existing PCT, HOMER, ML-FOREST, BARF-MLC, and FSEA-MLC models of the thyroid dataset in terms of accuracy, precision, recall, and f-measure are provided in Table 6. From the above table, it is proven that the proposed ASD-MLC obtains better results in all metrics, such as accuracy, precision, recall, and f-measure, than the existing PCT, HOMER, ML-FOREST, BARF-MLC, and FSEA-MLC models. For example, the proposed ASD-MLC achieves 90.07% accuracy, and the existing PCT, HOMER, ML-FOREST, BARF-MLC, and FSEA-MLC models achieve 67.44%, 71.56%, 83.55%, 86.42%, and 88.12%.

**Figure 3:**
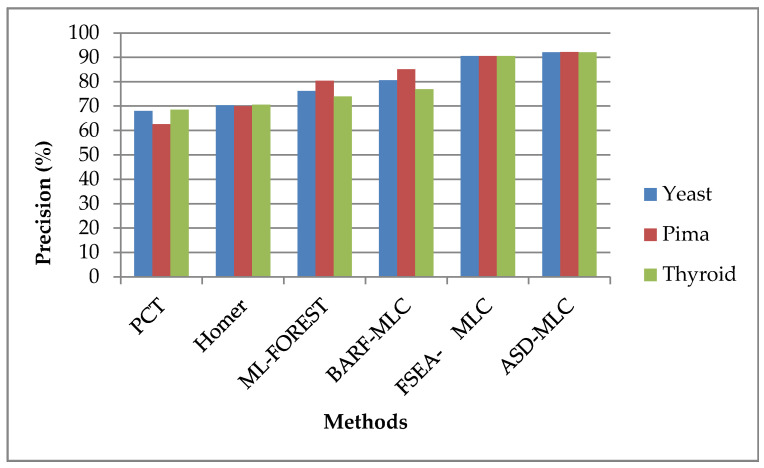
Precision is the basis for comparing different classifiers.

Figure 3 illustrates a comparison of several classifiers based on their accuracy as a percentage. The classifiers are applied to three different datasets, namely, Pima Indians, yeast 1, and thyroid. The fitness function of the IPSO shows improved optimization so that it improves the true-false rate of the suggested system. From the outcomes, it is concluded that the suggested ASDMLC technique has a higher precision value; for example, while the dataset Pima Indians proposed ASDMLC produces a higher precision value, which is 92.1362%, whereas other methods such as PCT, HOMER, ML-FOREST, BARF-MLC, and FSEA-MLC. Classifiers produce only 62.5456%, 69.9787%, 80.4444%, 85.0940%, and 90.5682% values, respectively.

**Figure 4:**
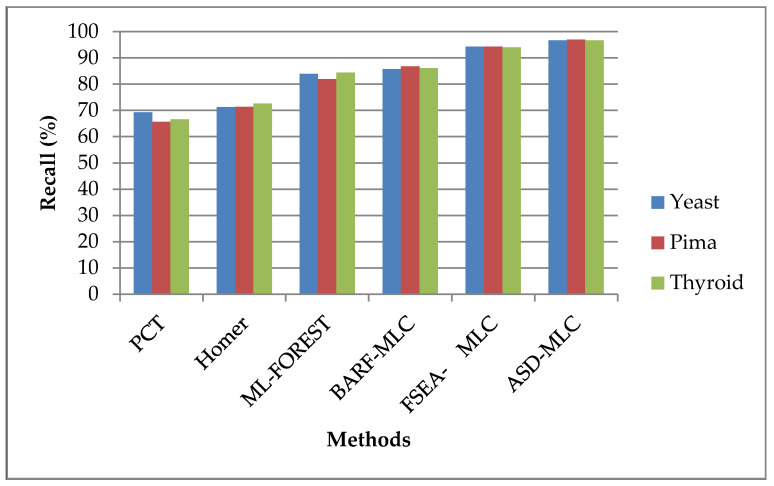
Comparison of the recall of different classifiers.

Performance comparison on various classifiers is based on recall in terms of percentage is shown in Figure 4. The classifiers are applied to three diverse kinds of datasets, namely, Pima Indians, yeast 1, and thyroid. According to the findings, the suggested ASDMLC approach has a greater recall value. For example, while the dataset was Pima Indians, the proposed ASDMLC produces a higher recall value which is 96.9539%, whereas other methods, such as PCT, HOMER, ML-FOREST, BARF-MLC, and FSEA-MLC classifiers produce only 65.6574%, 71.3579%, 81.8965%, 86.7701%, and 94.3286% values, respectively. 

**Figure 5:**
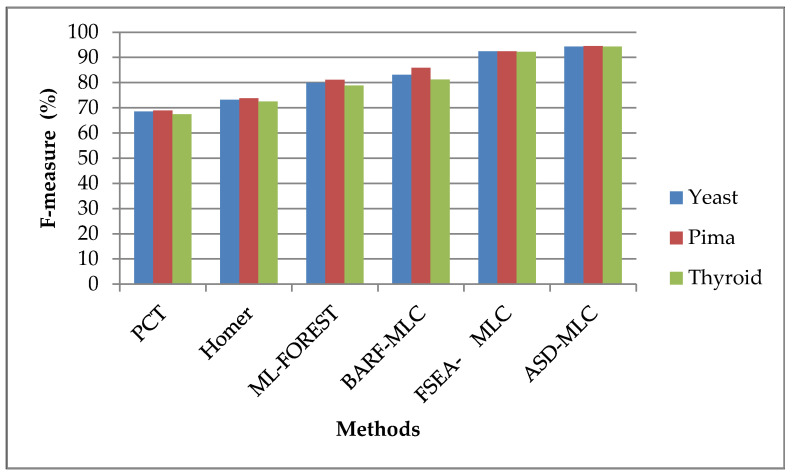
F-measure comparison of different classifiers.

In comparing the suggested method’s performance to that of the current classifiers according to the f-measure, the suggested technique’s performance is shown in Figure 5. The classifiers are applied to the various kinds of datasets, namely, Pima Indians, yeast 1, and new thyroid 1. The analysis of the data led to the conclusion that the suggested ASDMLC approach has great f-measure value. For example, while the dataset was Pima Indians, the proposed ASDMLC produces a higher f-measure value which is 94.4836%, whereas other methods, such as PCT, HOMER, ML-FOREST, BARF-MLC, and FSEA-MLC classifiers produce only 68.8955%, 73.7899%, 81.1650%, 85.9239%, and 92.4101% values, respectively. 

**Figure 6:**
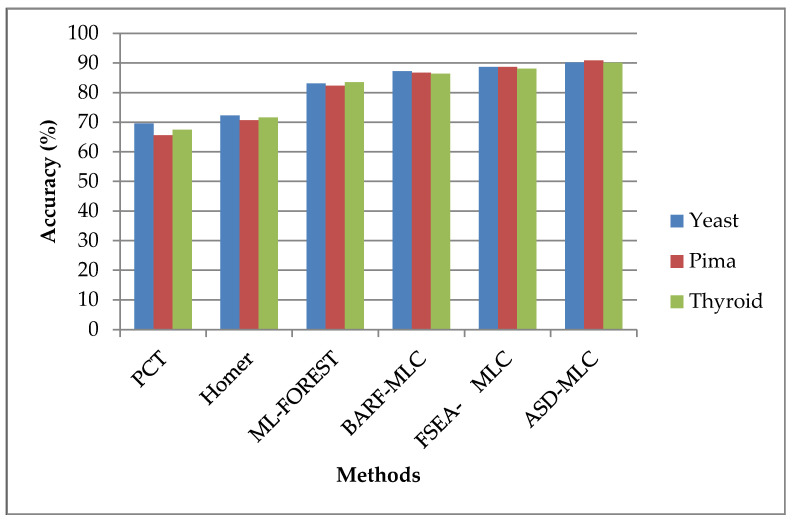
Comparison of the accuracy of different classifiers.

Figure 6 displays the accuracy-based efficiency of several classifiers as a percentage. The classifiers are used with three separate datasets: Pima Indians, yeast 1, and thyroid. When using the ASDMLC approach, minority class examples are distributed according to how difficult they are to learn, with the harder-to-learn examples receiving more synthetic data, which increases the accuracy of the classifier. The analysis of the data led to the conclusion that the suggested ASDMLC approach has a great accuracy value. For example, while the dataset was Pima Indians, the proposed ASDMLC produces a higher accuracy value which is 90.8854%, whereas other methods, such as PCT, HOMER, ML-FOREST, BARF-MLC, and FSEA-MLC, classifiers produce only 65.5667%, 70.6567%, 82.2917%, 86.7188%, and 88.6719% values, correspondingly. 

Table 7 shows the performance comparison results of the ASDMLC model with and without VPSO in terms of Accuracy, Precision, Recall, and F-measure. 

**Figure 7:**
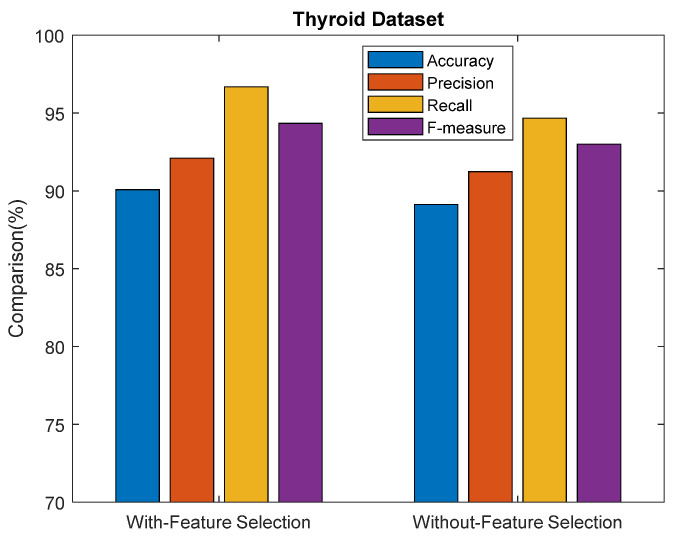
Comparison results of the proposed ASDMLC model with and without VPSO for the thyroid dataset.

Figure 7 shows the performance comparison results for the proposed ASDMLC model with VPSO and ASDMLC without VPSO in terms of accuracy, precision, recall, and f-measure for the thyroid dataset. From the results, it is concluded that the proposed ASDMLC model with VPSO achieves better results than ASDMLC without VPSO in terms of all metrics. For example, the ASDMLC model with VPSO produces 90.07% accuracy, and the ASDMLC without VPSO produces 89.12%.

**Figure 8:**
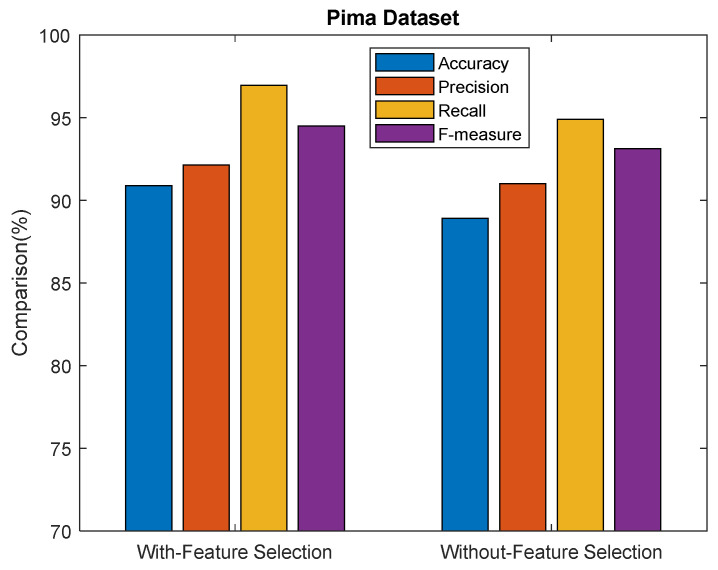
Comparison results of the proposed ASDMLC with and without VPSO for the Pima dataset.

The above Figure 8 shows the performance comparison results for the proposed ASDMLC model with VPSO and ASDMLC without VPSO in terms of accuracy, precision, recall, and f-measure for the Pima dataset. From the results, it is concluded that the proposed ASDMLC model with VPSO achieves better results than ASDMLC without VPSO in terms of all metrics. For example, the ASDMLC model with VPSO produces 90.88% accuracy, and the ASDMLC without VPSO produces 88.90%. 

**Figure 9:**
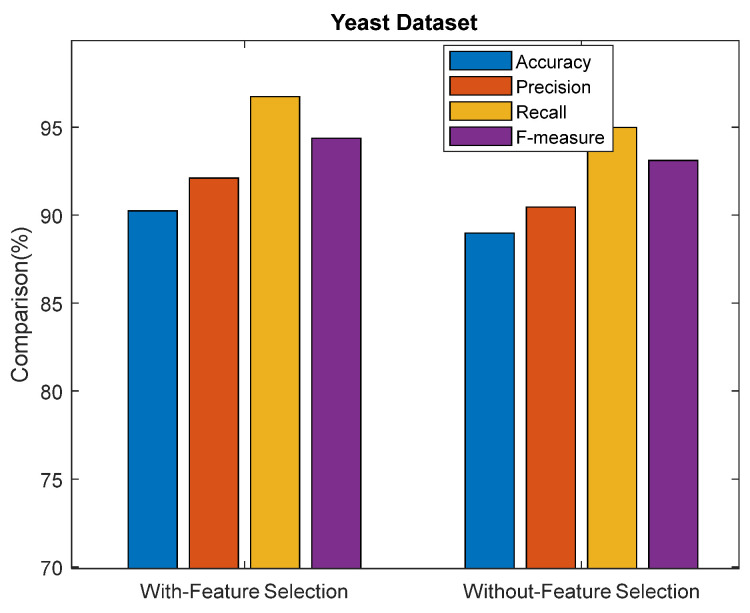
Comparison results of the proposed ASDMLC with and without VPSO for the yeast dataset.

Performance comparison results for the proposed ASDMLC model with VPSO and ASDMLC without VPSO in terms of accuracy, precision, recall, and f-measure for the yeast dataset are shown in Figure 9. From the results, it is concluded that the proposed ASDMLC model with VPSO achieves better results than ASDMLC without VPSO in terms of all metrics. For example, the ASDMLC model with VPSO produces 90.23% accuracy, and the ASDMLC without VPSO produces 88.98%.

## 5. Conclusions and Future Work

In multi-label learning, each instance in the training set is associated with a set of labels, and the task is to output a label set whose size is unknown a priori for each unseen instance. This work presented an innovative scheme called Adaptive Synthetic Data-Based Multi-label Classification (ASDMLC). A sampling strategy for learning from unbalanced data sets is called ADAptive SYNthetic (ADASYN) sampling. To standardize the magnitude of each variable’s impact on the outcomes, min-max normalization is employed to normalize the numerical variables. Velocity equalized Particle Swarm Optimization is used for feature selection, which selects more relevant features from the samples to increase the accuracy achieved with multi-label classification. To uncover the inherent label dependencies, the Ensemble Method for Multi-Label Classification (EM-MLC) will be processed in the end. Results show that the proposed system achieves 90.07% accuracy, 92.09% precision, 96.68% recall, and 94.33% f-measure for the thyroid dataset. Existing models, such as PCT, obtain 67.44% accuracy, 68.56% precision, 66.56% recall, and 67.43% f-measure. HOMER obtains 71.56% accuracy, 70.56% precision, 72.54% recall, and 72.54% f-measure. ML-FOREST achieves 83.55% accuracy, 73.94% precision, 84.36%recall, and 78.81% f-measure. BARF-MLC achieves 86.42% accuracy, 76.86% precision, 86.09% recall, and 81.21%f-measure. FSEA-MLC achieves 88.12% accuracy, 90.54% precision, 94.05% recall, and 92.26% f-measure. From the results, it is concluded that the proposed model achieves better performance than other existing models. However, the proposed model consumes more time for computation, so we need to use dimensionality reduction in future work and can use deep learning to further extend this model.

## Figures and Tables

**Table 1:** Comparative analysis of the existing approaches.

Author	Approaches	Discussion	Demerits
**Wang [13] et al. (2018)**	Multi-label Classification Utilizing an Improved Convolutional Neural Network Algorithm	It achieves an average labeling accuracy above 93%	Quite difficult the classification of images in various positions
**Jindal [15] (2018)**	A novel method for multi-label text document categorization that is both automatic and effective	Yields reasonable performance achieving an accuracy of 75%.	However, word embedding does not discriminate against different senses.
**Charte [16] et al. (2019)**	Tackling Multi-label Imbalance using Label Decoupling and Data Resampling Hybridization	Hamming Loss and Ranking Loss are minimized.	Performance degradation in using the high dataset
**Alyousef [17] et al. (2019)**	Identification of Illness Subclasses Using Latent Class Multi-label Classification for Better Prediction	Results show that the “Latent Class Multi-label Classification Model” increases the accuracy in comparison with contemporary potential techniques.	The primary disadvantage is that it is undesirable for datasets having a huge number of labels, owing to the massive exploration space
**Wang et al. [18] (2020)**	Active k-label sets ensemble	Feasible and effective	How to further improve the training efficiency will be an important issue
**Che et al. [19] (2021)**	FL-MLC	Is effective and diverse for multi-label classification	Increases the time complexity
**Sun et al. [20] (2021)**	Margin-based MNRS model	Effective and feasible	Increases the false positive rate
**Huang et al. [21] (2022)**	Correlation-based label thresholding	Produces better performance	Does not evaluated on high volume data
**Bayati et al. [22] (2022)**	Subspace learning and memetic algorithm	Superior to comparing methods	Increases the false positive rate
**Zhu et al. [23] (2023)**	Dynamic Ensemble learning	Outperforms the state-of-the-art methods.	Time-consuming nature
**Zhang et al. [24] (2023)**	Relief-LIFT	Achieve better performance	This does not apply to all applications

Existing works presented in the above literature review do not use proper feature-selection methods to reduce the time complexity, and they do not use any oversampling models to balance the imbalanced class problem in the data samples. To avoid those problems, the proposed work used enhanced feature-selection and data oversampling models, which increases the classification accuracy and reduces the time complexity.

**Table 2:** Information about imbalanced dataset.

Name of the Data Set	Attributes (Real/ Integer/Nominal)	Example	Imbalance Ratio
Pima Indians Data Set	8 (8/0/0)	768	1.87
Yeast 1 Data Set	8 (8/0/0)	1484	2.46
New—thyroid 1 Data Set	5 (4/1/0)	215	5.14

Pima Indians, yeast, and thyroid datasets are the three categories of data used in this paper. The objective is to forecast Pima Indian diabetes five years in advance using machine learning. A particularly effective framework that could do this would aid in targeting the afflicted with preventative actions. The description of the whole dataset may be found at the link.

**Table 3:** Experimental Setup.

SRL Num	Software Component	Component Description
1.	Coding Language	MATLAB 2013a
**SRL Num**	**Hardware Component**	**Component Description**
1.	System	Intel Core Processor
2.	Hard Disk	40 GB
3.	Floppy Drive	44 Mb
4.	Monitor	15 VGA Colour
5.	Ram	512 Mb

**Table 4:** Performance comparison results for yeast dataset.

Metrics	Methods					
	PCT	Homer	ML-F	BARF-MLC	FSEA-MLC	ASD-MLC
**Accuracy**	69.53	72.26	83.07	87.23	88.67	90.23
**Precision**	67.98	70.34	76.23	80.59	90.56	92.10
**Recall**	69.32	71.23	83.93	85.71	94.32	96.73
**F measure**	68.56	73.15	79.90	83.07	92.41	94.36

**Table 5:** Performance comparison results for Pima dataset.

Metrics	Methods					
	PCT	Homer	ML-F	BARF-MLC	FSEA-MLC	ASD-MLC
**Accuracy**	65.56	70.65	82.29	86.71	88.67	90.88
**Precision**	62.54	69.97	80.44	85.09	90.56	92.13
**Recall**	65.65	71.35	81.89	86.77	94.32	96.95
**F measure**	68.89	73.78	81.16	85.92	92.41	94.48

**Table 6:** Performance comparison results for thyroid dataset.

Metrics	Methods					
	PCT	Homer	ML-F	BARF-MLC	FSEA-MLC	ASD-MLC
**Accuracy**	67.44	71.56	83.55	86.42	88.12	90.07
**Precision**	68.56	70.56	73.94	76.86	90.54	92.09
**Recall**	66.56	72.54	84.36	86.09	94.05	96.68
**F measure**	67.43	72.54	78.81	81.21	92.26	94.33

**Table 7:** Performance comparison results of the ASDMLC model with and without VPSO.

Dataset	Metrics	Methods	
		ASDMLC	ASDMLC + VPSO
Thyroid	Accuracy (%)	89.12	90.07
	Precision (%)	91.23	92.09
	Recall (%)	94.67	96.68
	F-measure (%)	93	94.33
Pima	Accuracy (%)	88.90	90.88
	Precision (%)	91	92.13
	Recall (%)	94.89	96.95
	F-measure (%)	93.12	94.48
Yeast	Accuracy (%)	88.98	90.23
	Precision (%)	90.45	92.10
	Recall (%)	96.73	94.98
	F-measure (%)	93.10	94.36

## Data Availability

There are no available data to be stated.
